# A Novel Model for Landslide Displacement Prediction Based on EDR Selection and Multi-Swarm Intelligence Optimization Algorithm

**DOI:** 10.3390/s21248352

**Published:** 2021-12-14

**Authors:** Junrong Zhang, Huiming Tang, Dwayne D. Tannant, Chengyuan Lin, Ding Xia, Yankun Wang, Qianyun Wang

**Affiliations:** 1 Faculty of Engineering, China University of Geosciences, Wuhan 430074, China; zjr@cug.edu.cn (J.Z.); chengyuanlin@cug.edu.cn (C.L.); cug_xia@cug.edu.cn (D.X.); 2 Three Gorges Research Center for Geohazards of Ministry of Education, China University of Geosciences, Wuhan 430074, China; wangqianyun@cug.edu.cn; 3 Badong National Observation and Research Station of Geohazards, China University of Geosciences, Wuhan 430074, China; 4 School of Engineering, University of British Columbia, Kelowna, BC V1V 1V7, Canada; dwayne.tannant@ubc.ca; 5 School of Geosciences, Yangtze University, Wuhan 430100, China; ykwang@yangtzeu.edu.cn

**Keywords:** landslide displacement prediction, complete ensemble empirical mode decomposition (CEEMD), edit distance for real sequence (EDR), multi-swarm intelligence (MSI), support vector regression (SVR)

## Abstract

With the widespread application of machine learning methods, the continuous improvement of forecast accuracy has become an important task, which is especially crucial for landslide displacement predictions. This study aimed to propose a novel prediction model to improve accuracy in landslide prediction, based on the combination of multiple new algorithms. The proposed new method includes three parts: data preparation, multi-swarm intelligence (MSI) optimization, and displacement prediction. In the data preparation, the complete ensemble empirical mode decomposition (CEEMD) is adopted to separate the trend and periodic displacements from the observed cumulative landslide displacement. The frequency component and residual component of reconstructed inducing factors that related to landslide movements are also extracted by the CEEMD and *t*-test, and then picked out with edit distance on real sequence (EDR) as input variables for the support vector regression (SVR) model. MSI optimization algorithms are used to optimize the SVR model in the MSI optimization; thus, six predictions models can be obtained that can be used in the displacement prediction part. Finally, the trend and periodic displacements are predicted by six optimized SVR models, respectively. The trend displacement and periodic displacement with the highest prediction accuracy are added and regarded as the final prediction result. The case study of the Shiliushubao landslide shows that the prediction results match the observed data well with an improvement in the aspect of average relative error, which indicates that the proposed model can predict landslide displacements with high precision, even when the displacements are characterized by stepped curves that under the influence of multiple time-varying factors.

## 1. Introduction

Landslides reactivated by the impoundment of a reservoir or rainfall can cause catastrophic losses such as casualties, road burying, and house damages, which seriously threaten the property and life safety of human society [1,2]. In 2019, there were approximately 6181 geological hazard events in China, causing economic losses of 2.77 billion yuan. Among these, 4220 were landslides, accounting for 68% of the geological hazards [3]. The development of more accurate and effective landslide displacement prediction methods is of great significance for the early warning of catastrophic landslide movements and is an active research area [4,5,6,7]. Through the information obtained from the prediction approaches, the landslide status can be evaluated, and the corresponding mitigation measures can be taken in advance to reduce the destructive effects of landslides.

Landslide prediction models can generally be divided into physical–mechanical and phenomenological models [8,9]. The physical–mechanical models are generally recognized as originating from the empirical formula proposed by Saito in 1965 [10], and a series of models have been developed based on creep theory in the following decades [11,12]. Owing to the complexity, strict application conditions, and time-consuming shortcomings of the physical–mechanical models, research on phenomenological models is becoming more and more popular nowadays [13]. By means of mathematical statistics and machine learning, measured landslide displacements are analyzed and modeled while considering the related factors, such as rainfall, the reservoir water level, groundwater level, etc., allowing for the prediction of landslide displacements [12,14].

The support vector machine (SVM) is a frequently used method among all phenomenological models. Nevertheless, when solving regression problems, the performance of the SVM model, also known as support vector regression (SVR), is highly influenced by the determination of penalty parameter C and kernel parameters g [15]. Therefore, research has focused on improving the predictive ability of SVR models for landslide displacements through optimization algorithms. In addition to some classical optimization algorithms such as the genetic algorithm (GA) [16], particle swarm optimization (PSO) [17,18,19], artificial bee colony (ABC) [20], and ant colony optimization (ACO) [21], recently, studies have advanced with the times, and some newly developed optimization algorithms start to be used [22,23]. Moreover, the continuous enhancement process of the optimization algorithm, as well as the evaluation of the prediction effect after using different frameworks, are also carried out at the same time. Miao et al. [24] adopted a variety of algorithms to optimize the SVR model and achieved a good application effect in the prediction of Baishuihe landslide displacement. Zhang et al. [25] made comparisons of the predictive capability of the SVR model optimized by ACO and GA and found the advantage of ACO-SVR with the consideration of the inducing factors’ frequency component. At present, the application of optimization algorithms on SVR-based landslide prediction model parameter optimization is limited. It is still necessary to apply new optimization algorithms to these SVR-based models and compare their performance in landslide prediction.

Although based on the no free lunch (NFL) theorem, any optimization algorithms are equivalent when their performance is averaged across all possible problems; the swarm intelligence optimization algorithms (SIs) still show competitive results in solving optimization problems [26]. Similar to evolutionary algorithms (EA) [27] and artificial neural network algorithms (ANN) [28], the SIs also belong to the nature-inspired metaheuristics method [29]. With its high robustness, the SIs have been applied in many fields, including data clustering, network traffic forecast, data classification, UAV control, etc. Liu et al. [30] proposed a model of a global artificial fish swarm algorithm optimized support vector regression (GAFSA-SVR) for the network traffic forecast; the simulation shows an improvement of forecast precision and is superior to GA and chaos particle swarm optimization (CPSO)-optimized SVR model. Ali et al. [31] adopted the ant lion optimization algorithm (ALOA) in optimal allocation and sizing of renewable distributed generation sources in various distribution networks and results confirmed the effectiveness of the proposed algorithm. Jiang et al. [32] proposed an opposition-based seagull optimization algorithm (OSOA) to overcome the shortage of classification models such as slow computation, instability, and sensitivity to noise. In this paper, six new SIs proposed after 2010, including the bat algorithm (BA) [33], grey wolf optimization (GWO) [34], dragonfly optimization algorithm (DA) [35], whale optimization algorithm (WOA) [36], grasshopper optimization algorithm (GOA) [37], and sparrow search algorithm (SSA) [38], have been tested and compared in the proposed model, and the most suitable optimization algorithm has been identified.

**Figure 1:**
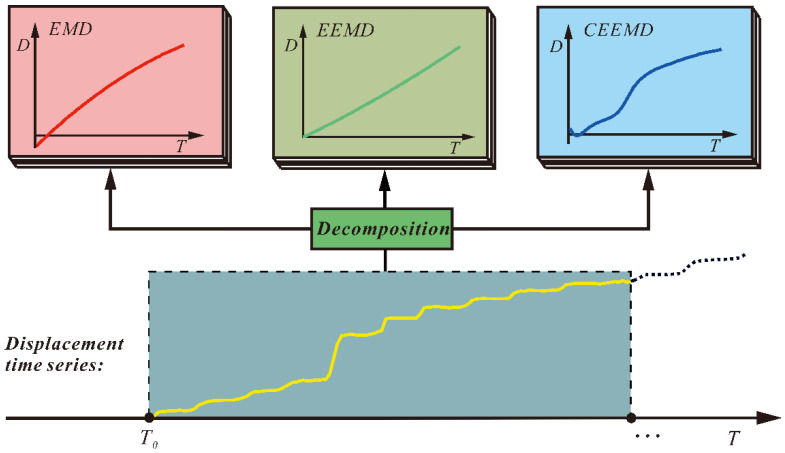
Residual terms of Baishuihe landslide displacements obtained through EMD, EEMD, and CEEMD.

Decomposition of landslide displacement is also a vital step in a prediction model and will directly affect the prediction effect. At present, decomposition method based on signal processing technology, for instance, Fourier transform (FT), discrete wavelet transforms (DWT), wavelet transform (WT), empirical mode decomposition (EMD), variational mode decomposition (VMD), and ensemble empirical mode decomposition (EEMD), are massively used in this field [39,40,41,42]. With these methods, the landslide displacement can be decomposed into a trend term and a periodic term, and then these components of the displacement can be predicted by different models. However, when using the CEEMD (complete ensemble empirical mode decomposition), the residual term shows a trend of first decreasing and then increasing, which is difficult to predict as a trend term compared with the residual terms of EMD and EEMD (Figure 1). Hence, a novel prediction model needs to be designed when the CEEMD is adopted in the decomposition of landslide displacement.

The screening of input parameters for an SVR model from related factors is an important part of prediction model optimization. Grey relational analysis (GRA) is a usual approach for this and has achieved convincing results [43]. Meanwhile, many other statistical methods such as maximal information coefficient (MIC) [23] and mean influence value (MIV) [44] have also been tried for this purpose. Zhang et al. [25] found that, as a similarity measuring method of time series, dynamic time warping (DTW) can be employed and works well in optimal input parameters selection of the SVR model. However, the DTW has the limitation of insensitive to the noise of the time series. To overcome this, the edit distance on real sequence (EDR) has been chosen and utilized in this study [45]. The EDR method is a classic trajectory similarity measurement that calculates the minimal number of editing operations needed for altering one sequence to another. With the advantages of robustness and accuracy, it has been utilized in traffic trajectory classification, physical movement similarity, and fiber segmentation, etc. [46]. It can also be applied in the related components selection for the prediction of landslides. Through calculating the similarity between restructured related factors sequence and periodic displacements sequence after normalization, two restructured related factors with minimum EDR value are the input variables of the SVR model.

This paper aims to improve the accuracy of landslide displacement prediction by constructing a novel model combined with the EDR method and multi-swarm intelligence (MSI). The new method can provide useful predictions of landslide displacements, allowing for the landslide status to be evaluated and the corresponding landslide mitigation measures to be taken before destructive movements occur.

In this paper, the next content is arranged as follows. In Section 2, the CEEMD, EDR, and MSI algorithms are briefly introduced. Section 3 considers the geological conditions and deformation features of the study case, the Shiliushubao landslide. The data preparation and statistical analysis of related factors are shown in Section 4. The predicted results and analysis are shown in Section 5. Section 6 discusses the proposed method, and conclusions are given in Section 7.

## 2. Methodology

### 2.1. Data Preprocessing with CEEMD

The CEEMD method is an effective improvement of the EMD method and EEMD method. By adding the white noise in the way of positive and negative pairs to the initial sequence of data, the residual auxiliary noise in the reconstruction signal can be better eliminated. Furthermore, the number of noise sets added can be very low, resulting in higher calculation efficiency. In CEEMD, based on local characteristics, the sequence can be converted to a limited number of intrinsic mode functions (IMF) and a residue. The operation of CEEMD includes three steps [47]:

Step 1: Add white noise consisting of positive and negative pairs to the original sequence data.

(1) $$\left[\frac{P}{T}\right]=\left[\begin{array}{cc} 1 & 1\\ 1 & -1 \end{array}\right]\left[\frac{\eta \left(t\right)}{N}\right]$$

where the original sequence is $\eta \left(t\right)$, *N* is the added white noise, and *P* and *T* are two reverse white noise. The number of the decomposed sequences is 2*n*, with *j* as the *j*th sample.

Step 2: Obtain a series of IMFs by decomposing *P* and *N* with the EMD method to generate two sets of IMFs.

(2) $$\left\{\begin{array}{c} P=\overset{m}{\underset{i=1}{\sum }}IMF_{ji}^{+}\\ T=\overset{m}{\underset{i=1}{\sum }}IMF_{ji}^{-} \end{array}\right.$$

where $IMF_{ji}^{+}$ is the *i*th IMF after adding the positive white noise, $IMF_{ji}^{-}$ is the *i*th IMF after adding the negative white noise, and *m* is the number of IMFs.

Step 3: Repeat step 1 and step 2 to get the corresponding IMF terms, and calculate the average of all the IMFs:

(3) $$IMF_{j}=\frac{\sum_{i=1}^{n}\left(IMF_{ij}^{+}+IMF_{ij}^{-}\right)}{2n}$$

Through this method, the original sequence can be expressed as the sum of some IMFs and a residue $r_{n}\left(t\right)$.

Zhang et al. pointed out that the CEEMD method combined with a *t*-test can obtain the high-frequency and low-frequency components from related factors such as rainfall and the reservoir water level through a fine-to-coarse reconstruction [25]. Moreover, according to the time series theory, the landslide displacement can be separated into a trend term and a periodic term by methods presented in the Introduction section. In this paper, the CEEMD is adopted as the decomposition method, and the obtained residual term is considered as the trend term. The result after the trend term is subtracted by the cumulative displacement of the landslide is regarded as the period term. Due to the special shape of the trend displacement time series after CEEMD decomposition, the displacement trend term and the period term will be predicted by the SVR model, respectively, later.

### 2.2. Selection of Optimal Related Factors via EDR

The EDR, which is based on Levenshtein distance, is a traditional and well-established similarity measurement method proposed by Chen et al. [45] and has been used for judging trajectory similarity since [48,49]. The EDR calculates the number of insertions, deletions, or replacement operations required to change the sequence R to T when the threshold is ε. It reduces the effect of noise by quantifying the distance into 0 and 1, and the Levenshtein distance method itself improves the local time-shifting situation (especially when the local time-shifting is not very large). Based on this, the displacement trend term sequence and residue of restructured related factors sequence were set as a reference sample sequence $R=\left\{r_{1},\;r_{2},\ldots ,\;r_{n}\right\}$ and a test sample sequence $T=\left\{t_{1},\;t_{2},\ldots ,\;t_{m}\right\}$ after normalization. Then, the EDR$\left(R,T\right)$ can be calculated as follows:

(4) $$match\left(r_{i},t_{j}\right)=true;if\left|r_{ix}-t_{jx}\right|\leq \varepsilon \;and\;\left|r_{iy}-t_{jy}\right|\leq \varepsilon$$

(5) $$D_{EDR}\left(R,T\right)=\left\{\begin{array}{c} n;\;if\;m=0\\ m;\;if\;n=0\\ Min\left\{\begin{array}{c} D_{EDR}\left(Rest\left(R\right),Rest\left(T\right)\right)+subcost,\\ D_{EDR}\left(Rest\left(R\right),T\right)+1,\;\;\;\;\;\;\;\;\;\;\;\;\;\;\;\;\;\;\;\;\;\;\;\;\;\;\\ D_{EDR}\left(R,Rest\left(T\right)\right)+1 \end{array}\right.;\;otherwise \end{array}\right.$$

(6) $$subcost=\left\{\begin{array}{c} 0,match\left(r_{1},t_{1}\right)=true\\ 1,otherwise\;\;\;\;\;\;\;\;\;\;\;\;\;\;\;\;\;\;\;\; \end{array}\right.$$

where the real number 0<ε<1 is the matching threshold. The cost for a replace, insert, or delete operation is set to 1. Therefore, through calculating the edit distance between two sequences, the smaller the EDR is, the greater the similarity will be. After calculating the EDR between the displacement trend term sequence and residues of original related factors sequence and restructured related factors sequence, three residues with the highest similarity were chosen as the input variable of the SVR model for predicting the displacement trend term.

Similarly, three optimal input variables for predicting the displacement periodic term with an SVR model can be obtained by calculating the EDR between displacement periodic term sequence and original related factors, restructured related factors and related factors frequency sequence.

### 2.3. Support Vector Regression (SVR)

The support vector regression (SVR) algorithm is a classic landslide displacement prediction model developed from statistical learning theory. With a powerful generalization ability and robust performance, the SVR model can easily solve quadratic programming problems with constraints. The main steps of an SVR model are summarized as follows [50].

Suppose that a nonlinear sample set in low dimensional space is: $\left\{x_{i},y_{i}\right\}$, where $x_{i}=\left\{x_{i1},x_{i2},\ldots ,x_{ip}\right\}$ is the input vector, $y_{i\;}$is the corresponding output vector, i is the number of samples and j is the number of input vectors. Then, the regression estimation function is:

(7) $$f\left(x\right)=w^{T}\varphi \left(x\right)+b$$

where w is the weight vector, $\varphi \left(x\right)$ is the nonlinear mapping function and b is the offset. Through minimizing the following equation, the value of w and b can be obtained:

(8) $$minJ=\frac{1}{2}\|w{\|}^{2}+C\overset{n}{\underset{i=1}{\sum }}\left(\xi_{i}^{+}+\xi_{i}^{-}\right)$$

(9) $$s.t.\left\{\begin{array}{c} y_{i}-w^{T}\varphi \left(x_{i}\right)-b\leq \varepsilon +\xi_{i}^{+}\\ w^{T}\varphi \left(x_{i}\right)+b-y_{i}\leq \varepsilon +\xi_{i}^{-}\\ \xi_{i}^{+},\xi_{i}^{-}\geq 0,i=1,2,...,n\;\;\;\;\;\; \end{array}\right.$$

where C and ε are the penalty parameter and the size of the insensitive loss function, respectively. $\xi_{i}^{+}\;\mathrm{and}\;\xi_{i}^{-}$ are the relaxation factors. By solving the quadratic optimization problem, the weight vector w can be expressed as:

(10) $$w=\overset{n}{\underset{i=1}{\sum }}\left(\beta_{i}^{*}-\beta_{i}\right)\varphi \left(x_{i}\right)$$

where $\beta_{i}^{*}\;and\;\beta_{i}$ are Lagrange multipliers. Therefore, the SVR model can be denoted as follows:

(11) $$f\left(x\right)=\overset{n}{\underset{i=1}{\sum }}\left(\beta_{i}^{*}-\beta_{i}\right)K\left(x_{i},x_{p}\right)+b$$

where $K\left(x_{i},x_{p}\right)$ is the kernel function. The SVR kernel function has various forms; in this study, the Gaussian radial basis function (RBF function) is chosen and adopted. Since algorithms for the determination of the penalty factor and the kernel function parameter (C, g) vary, the approach for selecting C and g must be further studied. Different forms of MSI algorithms were explored for the parameter optimization of the SVR model and all of them are briefly described next.

### 2.4. Multiple Swarm Intelligence

#### 2.4.1. Bat Algorithm (BA)

The bat algorithm (BA), proposed in 2010 by Yang et al., is a novel swarm intelligence optimization technique that simulates the echolocation behavior of microbats [33]. Based on iteration, this algorithm describes the echolocation of microbats and uses it to minimize any objective function and solve optimization problems. In BA, after initializing a group of random solutions, the optimal solution is searched by iteration, and a new local solution is generated by a random flight around the optimal solution, which strengthens the local search. BA is an accurate and effective method of finding the optimal parameter values for an SVR model with few parameters to adjust.

#### 2.4.2. Grey Wolf Optimization (GWO)

The grey wolf optimization (GWO) algorithm is a new swarm intelligent optimization algorithm proposed by Mirjalili et al. [23,51]. Based on the predatory behavior and strict social dominant hierarchy of grey wolves, this algorithm first randomly generates a group of gray wolves in the search space. Then, the wolves are divided into four social hierarchies according to the fitness from high to low, each marked with alpha, beta, delta, and omega. The location and distance between the grey wolves and the prey, which is the possible solution of the optimized SVR model, is obtained through iterative calculation. Finally, through the evolution of the wolf group itself, the distance between them is gradually reduced to realize the optimal hunting of prey. The algorithm has the advantages of strong convergence, few parameters, and easy implementation.

#### 2.4.3. Dragonfly Algorithm (DA)

The dragonfly optimization algorithm (DA) is a swarm intelligent optimization algorithm proposed by Mirjalili et al. [35,52]. The algorithm is based on the dynamic and static swarm behavior of dragonflies in nature, which includes separation behavior, alignment behavior, cohesion behavior, foraging behavior, and distraction from enemy behavior. By establishing a mathematical model of all these behaviors, the dragonfly’s latest position vector, which is a possible solution of the objective function, is calculated. This algorithm has the advantages of simple calculation, low complexity, few control parameters, and fast convergence speed.

#### 2.4.4. Whale Optimization Algorithm (WOA)

The WOA algorithm is a new heuristic optimization algorithm. The key idea is to simulate the behavior of humpback whales [36]. The humpback whales hunt in a special way using bubble nets, which can be described as two mechanisms: upward spirals and double loops. The WOA optimization algorithm has three steps: searching and encircling prey, the bubble-net argument attacking method (exploitation phase), and search for prey (exploration phase). Through this, the position vector of humpback whales with the best fitness value can be obtained by satisfying a termination criterion, and the final position vector is chosen as the best solution of the optimized SVR model parameters. The algorithm has the advantages of simple operation, few parameters to adjust, and a strong ability to jump out of a local optimum.

#### 2.4.5. Grasshopper Optimization Algorithm (GOA)

The grasshopper optimization algorithm (GOA), proposed by Saremi et al., in 2017, is a metaheuristic bionic optimization algorithm that mimics the swarming behavior of grasshoppers during population migration (exploration) and foraging behavior (exploitation) [37]. The grasshoppers’ position vector is equal to the value of an objective function [53]. When the grasshoppers reach a food source, the parameters reach the optimal variable, and the optimal value of the SVR model parameters is obtained. The algorithm provides a balanced condition between local and global search operators to achieve the final target. Two forces in grasshoppers, attraction and repulsion, provide global search and local search, respectively. To obtained effective solutions, the influence of the grasshopper’s current position, its relative position to other grasshoppers, and the position of the target point are regarded as the effective agents to determine the search vector. It has higher search efficiency and faster convergence speed, and its special adaptive mechanism can balance the global and local search processes with better optimization accuracy.

#### 2.4.6. Sparrow Search Algorithm (SSA)

The sparrow search algorithm (SSA), as proposed by Xue et al. [38], was mainly inspired by the foraging behavior and anti-predation behavior of sparrows. Some sparrows are in charge of seeking food and providing locations for the entire population, while the remaining sparrows use the locations to obtain food. Meanwhile, when a sparrow is aware of the danger and alarms, the entire population will immediately take anti-predation behavior. Although idealized, these behaviors are formulated with corresponding rules, and the algorithm classifying the sparrows into producers and scroungers. Their positions are updated according to their own rules, separately. In SSA, the position of each sparrow is equal to a possible solution of the objective function, and the best solution can be obtained when meeting iteration conditions. The algorithm is novel and has the advantages of a strong optimization ability, fast convergence speed, fewer adjustment parameters, and simple calculation.

### 2.5. Procedure of the Proposed Hybrid Algorithm

**Figure 2:**
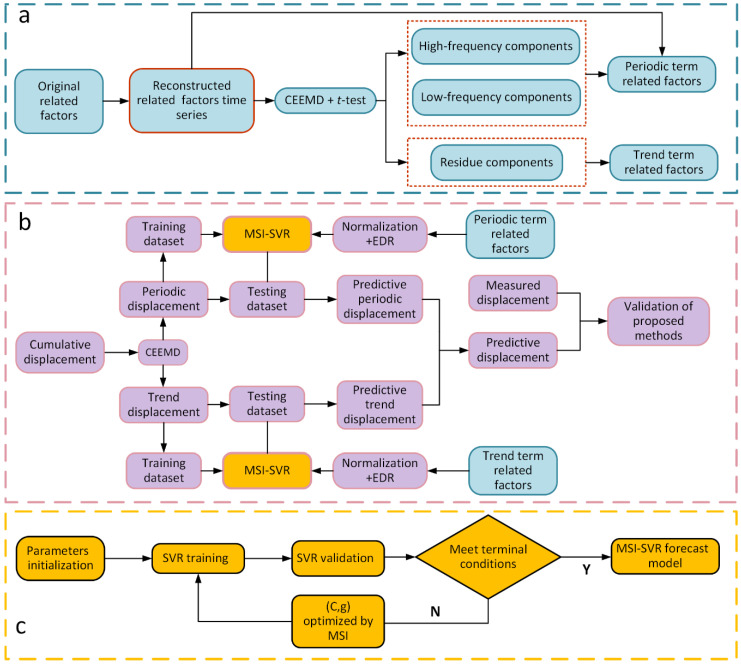
Framework of the proposed ensemble prediction model, (**a**) data preparation step, (**b**) displacement prediction step, and (**c**) MSI optimization step.

The framework of the proposed ensemble prediction model is shown in Figure 2. The entire forecasting process is divided into three steps: data preparation, multi-swarm intelligence (MSI) optimization, and displacement prediction. In the data preparation step, the time-sequences of factors related to the landslide movements, such as rainfall and reservoir water level, are restructured. The frequency component and residual component of all original and restructured sequences are then obtained through the combined application of CEEMD and *t*-test. In the MSI optimization step, MSI optimization algorithms are used to select the optimal C and g for the SVR model. In the displacement prediction step, the trend and periodic displacements are extracted from the observed cumulative landslide displacement through CEEMD. Then EDR is used to select the input variables of the periodic displacement prediction SVR model by calculating the EDR value between the periodic displacement and original related factors, restructured related factors, and frequency related factors after normalization. Similarly, the input variables of the trend displacement prediction SVR model are obtained by calculating the EDR value between the trend term displacement and all residue terms after normalization. Finally, the predictions of the trend and the periodic displacements are performed separately, and the total predicted displacement is obtained by adding them together.

### 2.6. Performance Evaluation Formula

The most commonly used indicators to evaluate the performance of prediction models are coefficient of determination (R^2^), root mean square error (RMSE), mean absolute error (MAE), and mean average percentage error (MAPE). These indicators were used in this study and are defined as:

(12) $$R^{2}=1-\frac{\sum_{i=1}^{N}{\left(y_{t}-{\hat{y}}_{t}\right)}^{2}}{\sum_{i=1}^{N}{\left(y_{t}-{\bar{\hat{y}}}_{t}\right)}^{2}}$$

(13) $$\mathrm{RMSE}=\sqrt{\frac{1}{N}\overset{N}{\underset{i=1}{\sum }}\left({\left(y_{t}-{\hat{y}}_{t}\right)}^{2}\right)}$$

(14) $$\mathrm{MAE}=\frac{1}{N}\overset{N}{\underset{i=1}{\sum }}\left|y_{t}-{\hat{y}}_{t}\right|$$

(15) $$\mathrm{MAPE}=\frac{1}{N}\left(\overset{N}{\underset{i=1}{\sum }}\left|\frac{{\hat{y}}_{t}-y_{t}}{y_{t}}\right|\right)\times 100\%$$

where $y_{t}$ is the $t^{th}$ measured value, ${\bar{y}}_{t}$ is the mean of the measured value, ${\hat{y}}_{t}$ is the $t^{th}$ predicted value, and ${\bar{\hat{y}}}_{t}$ is the mean value of the prediction.

## 3. Cases Study

### 3.1. Geological Conditions

**Figure 3:**
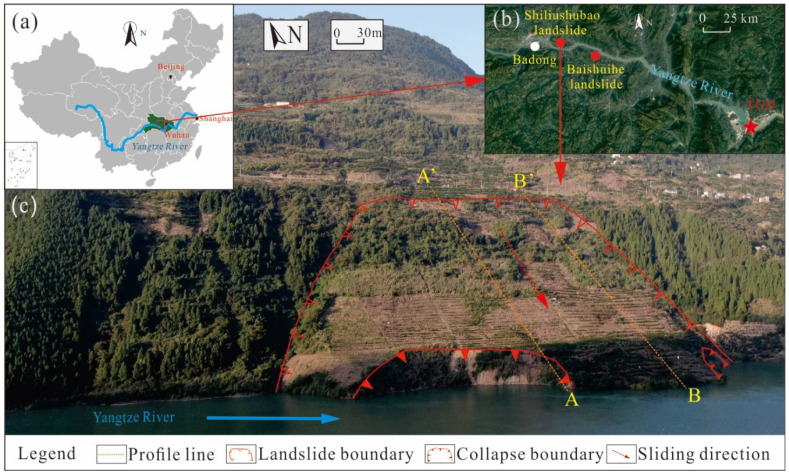
Location (**a**,**b**) and an oblique view (**c**) of Shiliushubao landslide captured by UAV, October 2020.

The Shiliushubao landslide is part of the famous Huanglashi landslide group, one of the large-scale landslides in the Three Gorges Reservoir Area (TGRA). It is located on the north bank of the Yangtze River, 1.5 km east of Badong county, 66 km away from the Three Gorges Dam (TGD) (Figure 3). The landslide’s geographical coordinates are 110°26′ east longitude and 31°02′ north latitude. The Shiliushubao landslide is bordered by the Lijiawan valley on the east and the Gan valley on the west, with a tongue-like shape. It is bigger than the well-known Baishuihe landslide with an estimated volume of 11.8 × 10^6^ m^3^ and covers an area of 0.34 km^2^. The top of the landslide is at an elevation of 340 to 358 m with a width of 140 m, and the toe of the landslide is at an elevation of 68 to 80 m with a width of 570 m.

**Figure 4:**
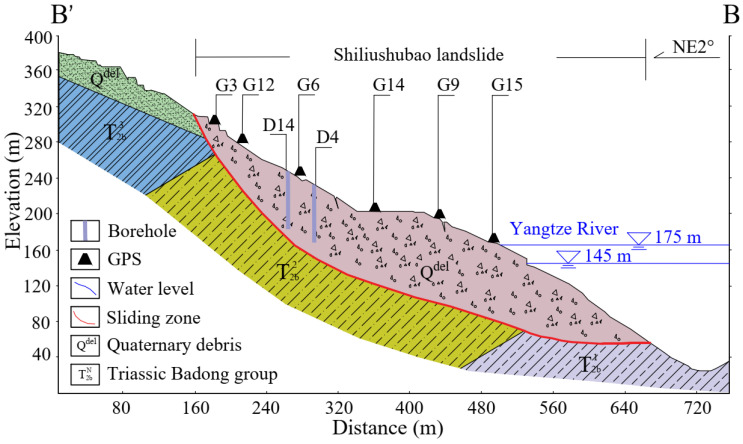
Geological section of the Shiliushubao landslide (B-B’).

The cross-section of the ground surface is shown in Figure 4 by the profile B-B’. The average slope angle is 26° along the sliding direction. However, the slope contains a gently sloping bench at an elevation near 200 m, and the slope is much steeper than 26° above and below the bench. The slope angle is up to 40° at elevations below the reservoir level.

The geological profile B-B’ in Figure 4. shows that the Shiliushubao landslide occurs in the Triassic Badong Group consisting of red mudstone, siltstone, gray-green marl, and limestone. These rocks are characterized by high clay mineral content (about 68%). Exposure of the rock to water allows the rock to soften and weaken. The sliding mass also includes near-surface Quaternary soils. The rear edge of the landslide is mainly a loose accumulation of gravel and clay. This soil is weak and is prone to collapses or sliding along the bedrock surface. The sliding zone consists of clay or silty clay with some gravel. The thickness of the sliding zone varies from 1.0 to 4.9 m, with an average thickness of 2.0 m.

**Figure 5:**
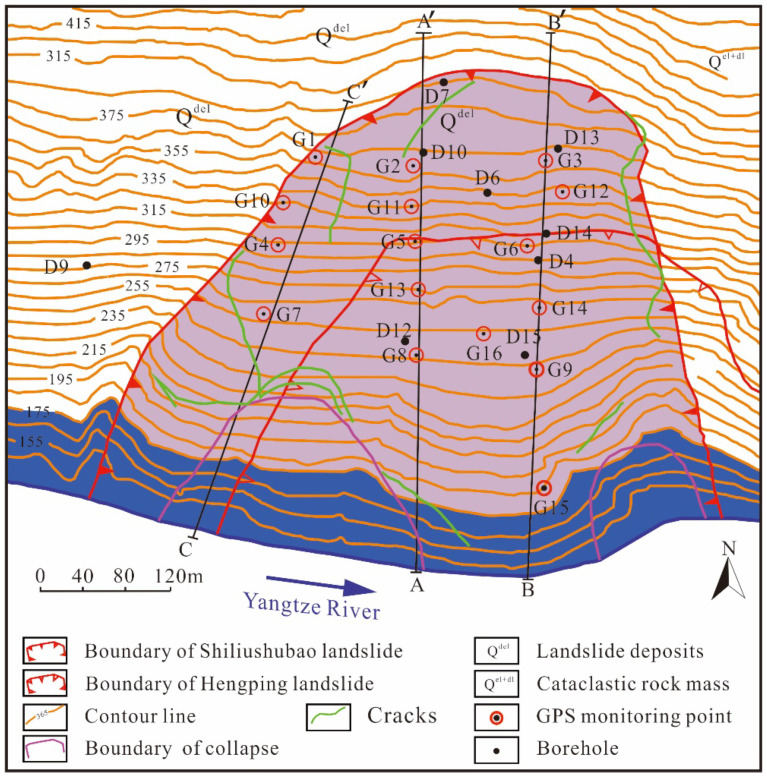
Geological map and monitoring points for the Shiliushubao landslide.

The topography of the lower part of the Shiliushubao landslide was mostly altered by the newly formed Hengping landslide (Figure 5), and some landslide materials under 100 m elevation have been removed by erosion. There are some small gullies near the landslide’s front edge caused by surface water runoff, which are the main channels for gathering and draining surface water.

### 3.2. Rainfall and Reservoir Levels

The Shiliushubao landslide is located in a subtropical zone, in which rainfall is continuous and concentrated in the summer. The rainy season generally occurs from May to September, which accounts for 70% of the yearly rainfall. Rainfall is one factor that increases the movement of the Shiliushubao landslide. Fluctuation in the reservoir level in the TGRA is another factor influencing the landslide movements, especially the sudden reservoir drops before the flood season.

### 3.3. Deformation Characteristics

**Figure 6:**
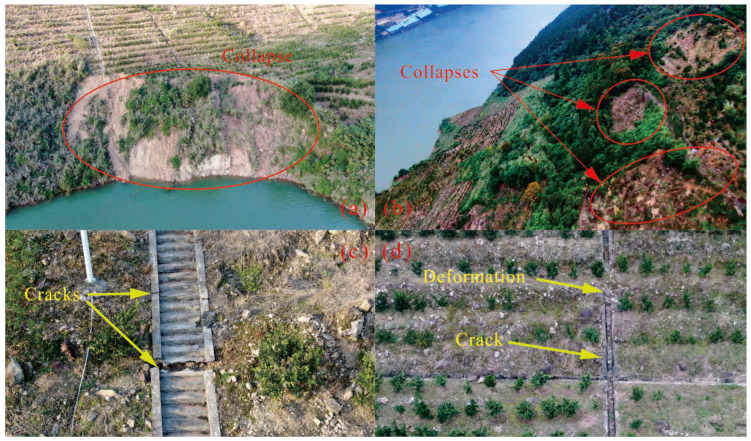
The ground collapses (**a**,**b**) and cracking (**c**,**d**) in the toe area captured by UAV, October 2020.

Since the reservoir was first impounded in June 2003, the toe area of the slope has experienced repeated small collapses (Figure 6). From 4 to 14 June 2004, four sliding events occurred during a period of rainfall, involving an estimated volume of 6000 m^3^. The toe area is very unstable, and slope movements at the toe affect the rest of the slope. At present, the slope’s deformation processes are causing small collapses under the influence of rainfall or reservoir level fluctuations.

Slope movements have created ground fissures that have gradually intensified. Areas of subsidence have also occurred. While the existing main cracks continued to expand, a series of new cracks gradually formed at the landslide’s rear edge. These cracks have connected and coalesced inside the sliding mass. The maximum crack length obtained by field monitoring is 345 m with opening widths up to 0.5 m and depths over 1 m. Many cracks have occurred in a concrete-lined drainage ditch at the front edge of the landslide. Moreover, some feathery cracks are also scattered along both sides of the landslide.

### 3.4. Landslide Monitoring

**Figure 7:**
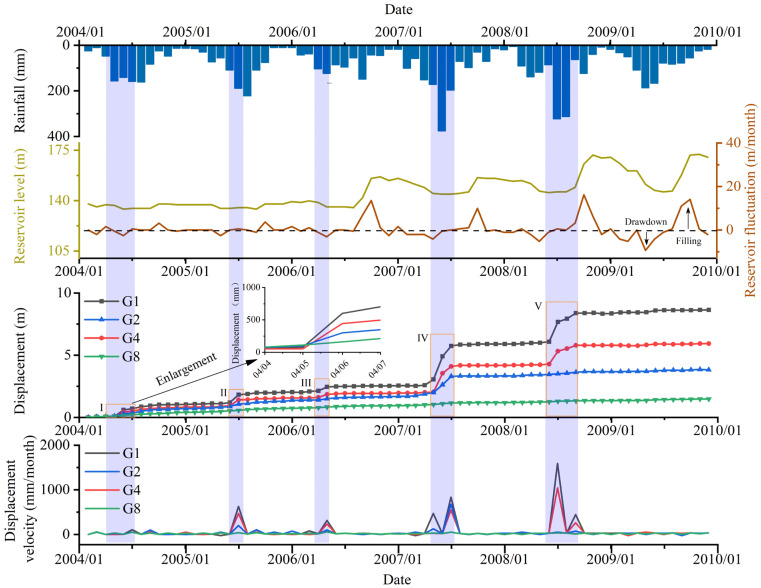
Displacement data from GPS points G1, G2, G4, G8, and rainfall and reservoir level data.

From February 2004 to December 2009, field monitoring was conducted to study the Shiliushubao landslide movements, based on which, the deformation evolution characteristics and development trend of the Shiliushubao landslide can be mastered. A total of sixteen GPS monitoring points and 15 boreholes were arranged on the surface of the sliding mass (Figure 5). Some monitoring points were destroyed due to rainfall, landslide movement, and other reasons. Thus, only monitoring data from February 2004 to December 2009 have been recorded and preserved. The cumulative displacement data from GPS points G1, G2, G4, G8, plus the rainfall and reservoir water levels were selected and shown in Figure 7.

### 3.5. Analysis of Monitoring Data

Monitoring data show that the displacement of the sliding mass increases with time in an obvious stepped shape. From February 2004 to December 2009, due to rainfall, the five displacement jumps occurred in the rainy season (May to September). After the rainy season, the landslide resumes movement at a slow, roughly constant speed.

The fluctuation of the reservoir water level is another factor affecting the deformation of the sliding mass. When the reservoir level drops sharply, the movement of the sliding mass accelerates. For example, from January to May 2007, the water level dropped from 155.4 m to 144.7 m, and the landslide displacement rate reached 16.2 mm/month in March when the water level dropped by 5 m. In May, when the water level dropped by 10.7 m, the landslide displacement rate was 44.4 mm/month. Similarly, when the reservoir water level fell in other periods, such as January to July 2009, the landslide displacement rate increased from 1.5 to 47.1 mm/month.

**Figure 8:**
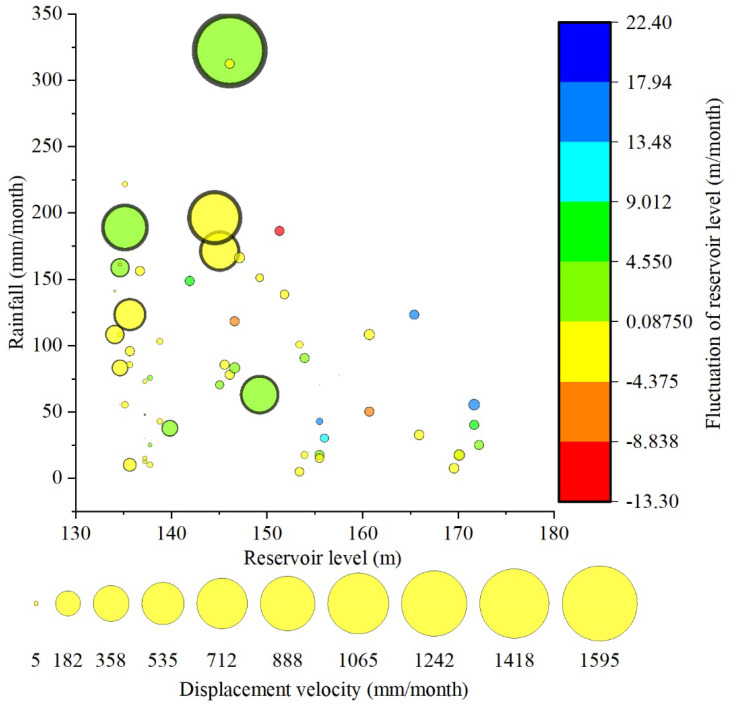
Correlation of displacement velocity at G1 versus the reservoir level, rainfall, and fluctuation of reservoir level.

For a better understanding of this seasonal deformation acceleration’s related factors, a correlation analysis between displacement velocity at G1 (located at the northeast edge of the landslide) and rainfall, rate of reservoir level change, and reservoir level are shown in Figure 8. The size of the bubbles represents the deformation velocity. The larger bubbles tend to plot where the rainfall is higher. Meanwhile, the large bubbles are mainly concentrated where the reservoir level is between 140 and 150 m and are located where the reservoir level fluctuates slowly (between −4.4 and 9.0 m/month). This indicates that reservoir level fluctuations mainly trigger accelerated landslide movements when the reservoir level is low. The maximum size bubble appears where the rainfall is about 325 mm/month, and the water level rises between 4.5 and 9.0 m/month. The combined effect of heavy rainfall and rising reservoir level on landslide deformation is more significant than low rainfall combined with reservoir level drawdown.

**Figure 9:**
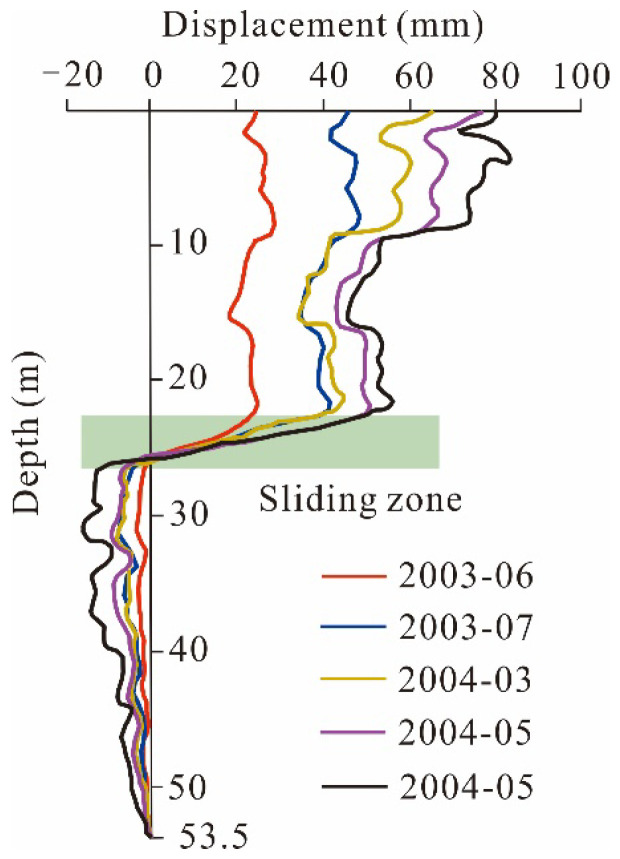
Lateral deformation versus depth in inclinometer D7.

Inclinometer D7 indicates that the main sliding zone is located at a depth of 22 to 26 m (Figure 9). The data show that before June 2003, the shear deformation in the slip zone was slow. Then, with the operation of the TGRA, the displacement in the shear zone increases. Therefore, it can be judged that the Shiliushubao landslide is in the stage of accumulative creep deformation, and the deformation tends to be intensified under the influence of reservoir water.

In conclusion, the formation of the Shiliushubao landslide is the result of a series of related factors including internal inducing factors and external inducing factors. Weak rock formations are the inherent cause of deformation. In the Badong Formation, soft rocks characterized by high clay mineral content account for about 68%. The hydrophilicity of the rock determines that the rock has the characteristics of easy softening, muddy and weathering, and lays the material foundation for the deformation and failure of the slope. Water is the external cause of deformation. The impact of concentrated high intensity rainfall and periodic water storage activities in the TGRA, especially the sudden drop before the flood season, are main external inducing factors for the reactive of the Shiliushubao landslide.

## 4. Data Processing and Statistical Analysis

### 4.1. CEEMD Decomposition of Landslide Displacement Versus Time Data

Since all displacements at the Shiliushubao landslide show a similar step-like deformation curve, only the displacement data at site G1 is chosen for model validation in this study. The CEEMD method can be used to extract the trend displacements and the periodic displacements. The following parameters were used [2]:

- ensemble member = 200
- standard deviation of added white noise in each ensemble member = 0.2
- threshold variance = 0.2
- threshold for first iteration = 4

The landslide displacement sequence was decomposed into a few IMFs and a residue through CEEMD. The residue is considered to be the trend displacement of the landslide, and the periodic displacement was obtained by adding all the IMFs together.

The results show that the trend displacement component of G1 has local fluctuations and an increasing trend over time, which is consistent with a long-term trend of cumulative displacements. The periodic displacement component shows a cyclical variation in displacements ranging from −800 to 917 mm. The maximum variation range of periodic displacement occurred in the 2007 rainy season when the TGR was first impounded. As the periodic displacement and trend displacement are important components of the cumulative displacement, they will be separately modeled and predicted. Once the best prediction for each component is obtained, the best prediction for cumulative displacement is obtained.

**Figure 10:**
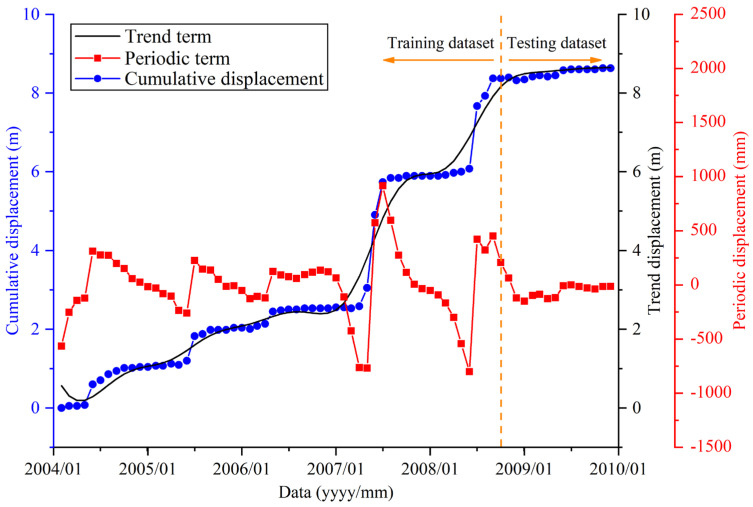
Periodic and trend displacement at site G1 obtained through CEEMD.

The displacement data are divided into training and testing data sets to establish the SVR prediction model of periodic and trend displacements (Figure 10). The SVR model is organized with the training dataset to establish the regression relationships between displacement and selected variables. The trained SVR model can then be used to predict the current month periodic displacement and compared with the testing dataset to verify the model’s accuracy. In this study, the displacement data from February 2004 to September 2008 were selected as the training dataset, and the rest were used as the testing dataset.

### 4.2. CEEMD Decomposition of Related Factors

Before selecting the input various parameters, the factors related to the landslide deformation are usually restructured first [24]. Original related factors such as the rainfall, reservoir level, and date of displacement were restructured. The current monthly rainfall sequence (L1) was restructured as the accrued precipitation of the previous two months (L2), as were the accrued precipitation of the previous month and the current month (L3), and the accrued precipitation of the previous two and the current month (L4). The current monthly reservoir level data (X1) were restructured as the reservoir level monthly change (X2) and the change of reservoir level between two months (X3). The displacement data (D) were restructured as the previous month displacement (D1) and the accrued displacement of the previous month and the current month (D2).

**Figure 11:**
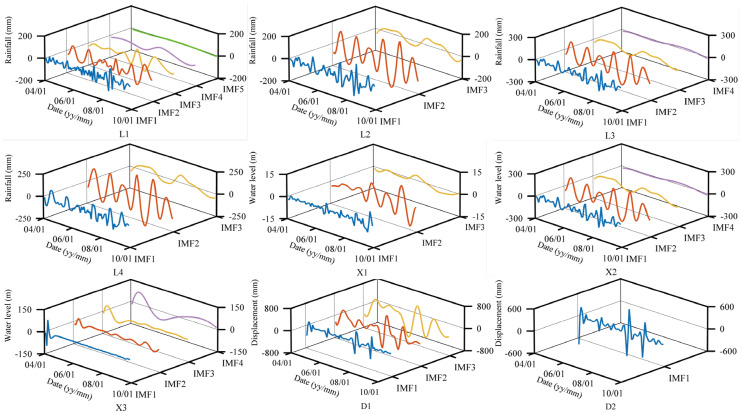
IMFs of restructured factors derived through CEEMD.

Keeping the CEEMD parameters fixed, L1–L4, X1–X3, and D1–D2, can be decomposed into a few IMFs sorted by frequency from highest to lowest and a residue. The mean of IMF_1_ was compared to the other IMFs by a paired *t*-test with a significance set at 0.05 (two-tailed) for each decomposed and restructured factor. If the significance values of IMF_i_ are greater than 0.05, the difference between IMF_1_ and IMF_i_ is not significant. Therefore, the superposition of IMFs from IMF_1_ to IMF_i_ is the high-frequency component, and the superposition of the remaining IMFs is the low-frequency component. The IMFs of each restructured factors are shown in Figure 11, and the results of the paired *t*-test are shown in Table 1.

The results reveal that the IMFs obtained from the decomposition of all factors show a certain periodicity. Their frequency varies, and IMF_1_ usually has the highest frequency and fluctuation amplitude. Since there is only one IMF after the CEEMD decomposition of D2, it is considered that there are only high-frequency components in D2. The paired *t*-test results indicate that only IMF_3_ in X1 and IMF_4_ in X3 has a significance value that is less than 0.05, which denotes that the low-frequency components only exist in X1 and X3. Taking these two as the low-frequency components of X1 and X3, the high-frequency components of the other factors will be the sum of the remaining IMFs. Therefore, in addition to the variables mentioned above, new variables can also be chosen as input to an SVR model of the periodic displacements after reconstruction: high-frequency current monthly rainfall sequence (L1^H^), high-frequency accrued precipitation of the previous two months (L2^H^), high-frequency accrued precipitation of the previous month and the current month (L3^H^), high-frequency accrued precipitation of the previous two and the current months (L4^H^), high-frequency current monthly reservoir level data (X1^H^), low-frequency current monthly reservoir level data (X1^L^), high-frequency reservoir level monthly change (X2^H^), high-frequency change of reservoir level between two months (X3^H^), low-frequency change of reservoir level between two months (X3^L^), high-frequency previous month displacement (D1^H^), and high-frequency accrued displacement of the previous month and the current month (D2^H^).

**Figure 12:**
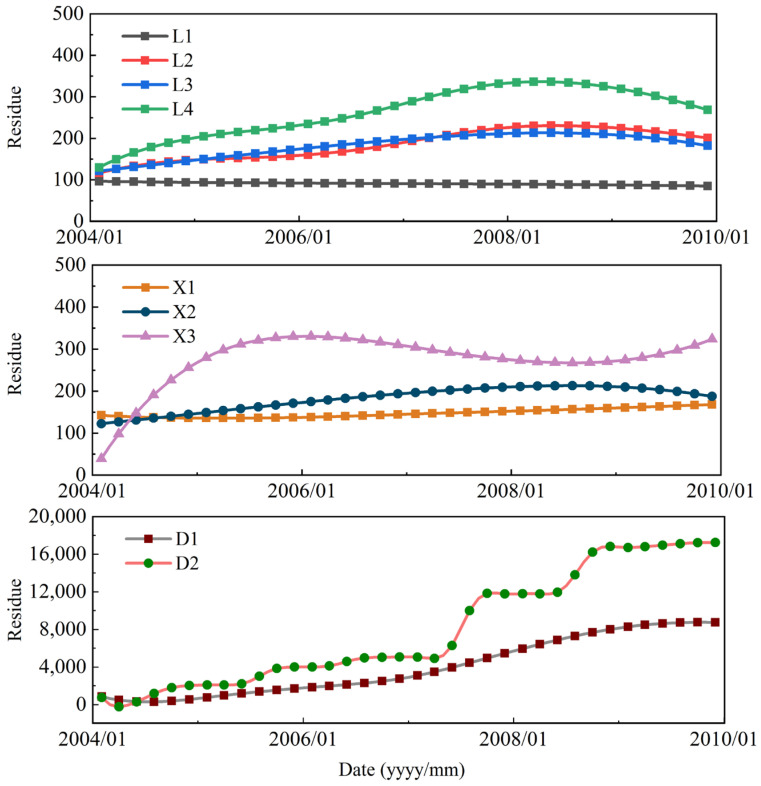
Residue term of restructured factors derived through CEEMD.

The residue terms of restructured factors derived through CEEMD are shown in Figure 12. The results demonstrate that, except for L1, all the residue terms show a roughly increasing trend that is similar to the trend displacement term. This suggests that the residual terms roughly reflect the trend of the cumulative displacement, allowing the residue terms to be used as input parameters for the SVR to predict the displacement trend term.

### 4.3. Factors Affecting Landslide Displacement Selected by EDR

Previous analyzes demonstrated a strong association between the landslide displacement and the aforementioned factors. Thus, it is vital to determine which factors that have the greatest influence on landslide displacement. The EDR distance was determined between each factor and the displacements to determine the specific factors most closely related to the landslide’s periodic displacement and trend displacement, respectively. This helps to identify the best factors to use the SVR model. The original restructured factors and their frequency components were chosen to compute the EDR distance with the periodic displacement. Simultaneously, the residue term for each factor and restructured factors were utilized to compute the EDR distance with the trend displacement. Normalization can be used to eliminate the influence of the numerical magnitude on analysis results due to the dimension difference between the displacement time series and the related factors. The calculated EDR distances are shown in Table 2.

After dividing all the related factors into rainfall, reservoir water level, and displacement groups, the factors with a smaller EDR distance can be regarded as more interrelated with the landslide displacement component in each group. The results show that, for periodic displacement, the high-frequency accrued precipitation of the previous two and current months (L4^H^), the high-frequency current monthly reservoir level data (X1^H^), and the high-frequency previous month displacement (D1^H^) are the most relevant factors in each group. Thus, when predicting periodic displacement, L4^H^, X1^H^, and D1^H^ are the input variables for the periodic displacement SVR model. Similarly, related factors for predicting trend displacement are the residual terms of L2, X1, and D2 according to the EDR results in each group, and these were chosen as the input parameters for the trend displacement SVR model.

**Figure 13:**
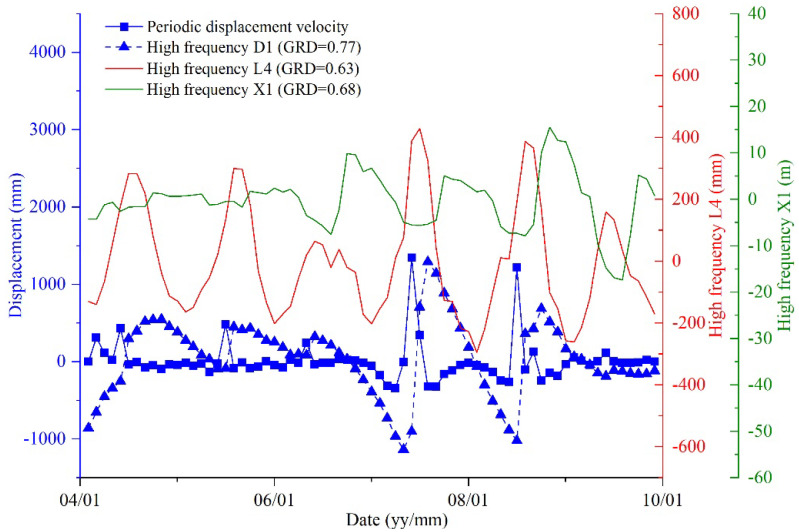
Landslide periodic displacement compared with selected factors affecting landslide movement.

To verify the effectiveness of the EDR method, grey relational analysis (GRA), a common method for selecting input variables in landslide displacement prediction, was used to calculate the grey relational degree (GRD) between the selected factors and the displacement component. The periodic displacement component is chosen as the research object, and the factor’s GRD and periodic displacement velocity are shown and compared in Figure 13. The factors with a GRD value higher than 0.6 are regarded as closely interrelated with the periodic displacement. Therefore, the high-frequency accrued precipitation of the previous two and current months (L4^H^), the high-frequency current monthly reservoir level data (X1^H^), and the high-frequency previous month displacement (D1^H^) are the most relevant related factors in each group, which is consistent with the results selected by EDR.

## 5. Prediction Results and Comparison

### 5.1. Parameter Optimization

**Figure 14:**
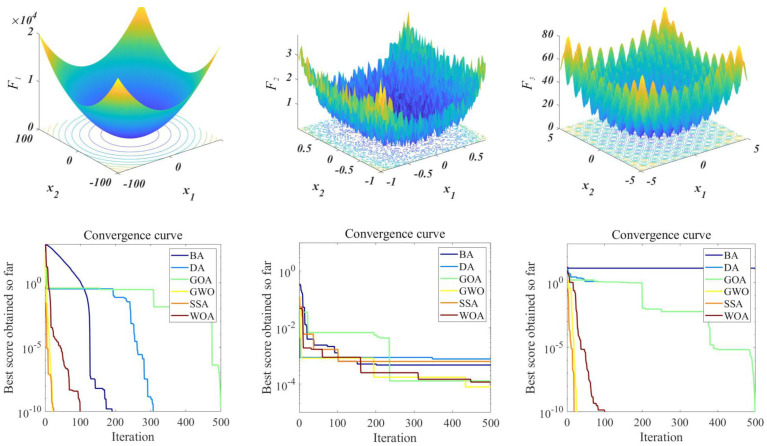
Iterative curves of three benchmark functions solved using multiple-SI.

For quantitatively measuring the optimization performance of the six SIs adopted in this study, three selected benchmark functions (Table 3) with different features are employed as test functions and results are shown in Figure 14. Different from $F_{2}\left(x\right)$ and $F_{3}\left(x\right)$, the $F_{1}\left(x\right)$ is smoother and has a unique extreme point in the solution space of *x*_1_ and *x*_2_. The calculation results and process show that the slopes of the convergence curves of SSA and GWO are close, indicating that the convergence performance of the two is close and is the best among the six algorithms. The solutions obtained by each SI in $F_{1}\left(x\right)$ and $F_{3}\left(x\right)$ are relatively scattered, and some algorithms (such as BA) will fall into a local optimum.

Determining the optimal value of the penalty factor C and the kernel function parameter g of the SVR model is a vital procedure dominating the accuracy of a displacement prediction. The parameters C and g in this study are optimized with MSI algorithms and are conducted independently for periodic and trend terms. For each MSI algorithm, the parameters C and g make a two-dimensional searching space. A population of simple agents communicate locally with each other and with their environment and move in specific patterns to search for the best result. The parameter settings and initial conditions in the MSI algorithm jointly affect the result. The parameter settings are iteratively adjusted and recalculated according to the optimal prediction effect. The results of the optimization are shown in Table 4. The optimized C and g are later used in the SVR-based model to predict the periodic and trend displacements.

### 5.2. Prediction of Periodic and Trend Displacements

**Figure 15:**
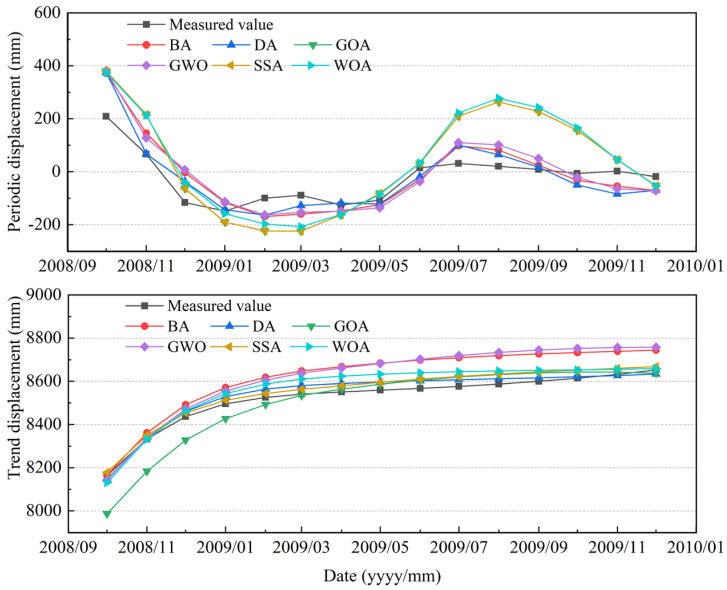
Comparison of prediction results by MSI-SVR model with monitoring data.

**Figure 16:**
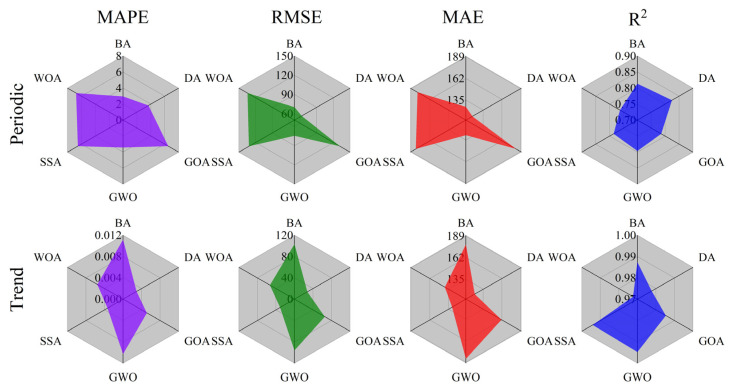
Rose diagram for each model’s performance.

An MSI-based SVR prediction model was developed with the optimized input factors to predict the periodic displacements and the trend displacements separately, as shown in Figure 15. The prediction accuracy and error of each model are shown and compared in Figure 16. For the periodic displacements, the prediction accuracy with the largest R^2^ and smallest MAPE, RMSE, and MAE was obtained using the DA algorithm among all of the given models. The corresponding result of MAPE, RMSE, MAE, and R^2^ is 3.654173, 63.0435, 119.2786, 0.824217, respectively. Meanwhile, the GWO-based SVR model gave the best prediction for the trend displacements compared to the other optimization algorithms, with the result of MAPE, RMSE, MAE, and R^2^ being 0.010273, 95.9178, 184.4194, and 0.99473, respectively. Overall, the prediction results provided by the SVR model optimized by MSI matched well with the observation results.

### 5.3. Prediction of Cumulative Displacements

**Figure 17:**
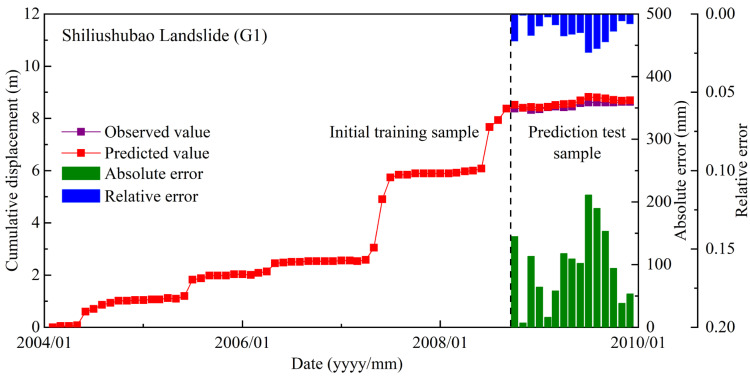
Comparison of predicted displacement and observed displacement.

The predicted cumulative displacements of the Shiliushubao landslide can be obtained by adding the predicted periodic and trend displacements. The predicted cumulative displacements are shown in Figure 17, and these are in good agreement with the observed displacements. The maximum relative error of monthly displacement is generally less than 3% and the average relative error of less than 1%. The results show the usefulness of the proposed model. The most appropriate optimization algorithm and the most relevant landslide related factors were selected and applied.

**Figure 18:**
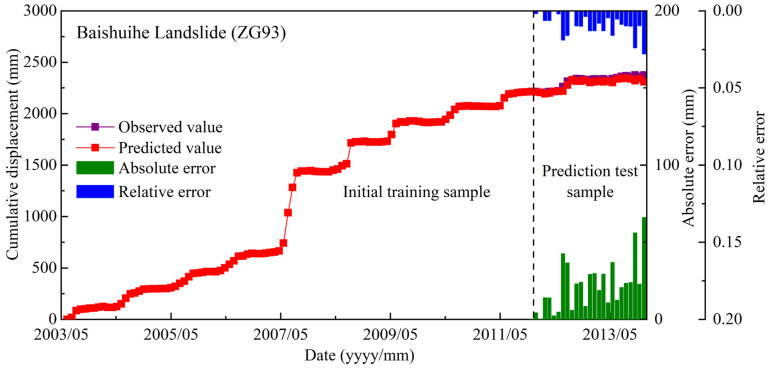
Comparison of predicted displacement and measured displacement.

To further verify the effectiveness of the proposed prediction model, the displacement at ZG93 of the well-known Baishuihe landslide is chosen as another case and predicted. The prediction accuracy of each SI and cumulative displacement prediction result are shown and compared in Table 5 and Figure 18.

The SSA method has achieved the best results in predicting both periodic displacement and trend displacement, with the largest value of R^2^, which is 0.762 and 0.9998, respectively. The cumulative displacement prediction results are in good agreement with the measured displacement, with an absolute error of monthly displacement that is generally less than 67mm and the maximum relative error of monthly displacement that less than 3%. The average relative error of the proposed prediction model is 0.898%, which is slightly smaller than the result obtained by the prediction model of Deng et al. [54]. The comparative study shows the effective improvement of the proposed model in terms of prediction performance and the universality of it to predict the displacement of slow-moving landslides all around the world.

## 6. Discussion

This paper aims to improve the accuracy of landslide displacement prediction by constructing a novel prediction model combined with the CEEMD method, EDR method, and multi-swarm-intelligence (MSI) algorithm. The new prediction model can forecast landslide movements so that the landslide status can be evaluated, and appropriate stabilization measures can be implemented in advance to reduce the destructive effects of landslide movements. The CEEMD method was first employed for the landslide displacement decomposition, and a new prediction based on this was proposed to overcome its defect by optimizing the model’s framework. The trend displacement obtained from CEEMD decomposition can reflect the long-term trend of landslide deformation. The periodic displacement obtained from CEEMD decomposition shows a cyclical variation in displacements consistent with periodic changes of related factors such as rainfall and reservoir levels. The frequency components of related factors that change periodically can be decomposed by the CEEMD. Combining with the *t*-test, the high-frequency and low-frequency components of related factors can be separated. With the EDR method, the most relevant factors related to the landslide displacements among the original related factors, reconstruction related factors, and frequency related factors can be selected by calculating the distance between all related factors and the extracted displacement component. The relevant factors that were identified are consistent with the results obtained by GRA.

The factors related to landslide displacement prediction can be separated into three groups: rainfall, reservoir level, and previous displacement. The most relevant factors for the Shiliushubao landslide’s periodic displacement are L4^H^, X1^H^, and D1^H^, and the most relevant factors for the trend displacements are the residual terms of L2, X1, and D2. MSI (BA, DA, GOA, GWO, SSA, and WOA) was used to optimize the proposed prediction model. For the Shiliushubao landslide, the DA-based SVR model performs best to predict periodic displacements, and the GWO-based SVR model works best for predicting trend displacements. The prediction of cumulative displacements is in good agreement with the measured displacements with a maximum relative error of monthly displacement of less than 3%. The trail of the proposed model on the Baishuihe landslide, another landslide in the reservoir area, is also satisfied with the average relative error of 0.898%, which performs slightly better than that from the previous study.

While the proposed methodology yielded satisfactory results, there are also some limitations. First, the CEEMD method has limits in the decomposition of measured displacements and related factors when the time series does not have enough extreme points, which limits the applicability of this method. When there is only one IMF sequence after CEEMD decomposition, a *t*-test cannot be carried out, and the IMF itself is high frequency. Second, the trend displacement and residue term for related factors after CEEMD decomposition may still have local fluctuations. It might contain some periodic fluctuation information, which can lead to prediction error, which needs to be further studied in the future. Third, the values of thrsh, sthresh, N, and alpha used in CEEMD will have an indirect impact on the prediction results. The appropriate range of these parameters and their influence on the results are still unclear. Fourth, when using MSI to optimize the parameters of the SVR model, the search for the g value is usually close to the lowest value of the search interval, and the result does not gradually increase as the search boundary continues to widen. Different SI optimization algorithms may perform differently for different landslides, so for new landslide data, the trial of different optimization algorithms for the best results is needed.

The deformation and failure of landslides are usually closely bonded with the groundwater effect [55]. The evaluation of the landslide stability with groundwater nowadays has developed into several hotspot branches, which includes analytical methods, such as the Limit Equilibrium Analysis with the Reliability Analysis and the Intelligent Algorithms on sliding zone searching, and numerical methods, such as the Fast Lagrange Analysis, the Finite Element Method, and the Discrete Element Method coupled with hydraulic calculations. These advanced evaluation methods have their status in the practical industry on the slope stability and deformation assessment, based on the current state and data gathered in the field and laboratory; however, these mechanism-based methods took insufficient account of the history state and data of the slope. The novel prediction model proposed in this paper can consider the historical influence of rainfall and reservoir fluctuation that precisely related to the displacement periodic component and displacement trend component with the help of the CEEMD method and EDR, thus improving the accuracy of landslide displacement prediction. It is a profitable attempt and a good way to improve the accuracy of landslide movement prediction. Although some in-depth research in consideration of historical factors of inducing factors has been carried out in this study, the predictive capability of the proposed model is still flawed in the sense that they cannot say anything about changes that are caused by external factors not captured by the available data series. Therefore, it is very important to develop multi-field (displacement field, seepage field, stress field, etc.) monitoring technology for the landslides, and the innovative prediction models based on this can more reflect the evolution process of the sliding mass.

Landslides in complex water environments could develop different deformation patterns, both categorized by history data and potential failure mechanism [4]. The pattern is highly related to the interaction between soil and water in a certain engineering geological condition. In the proposed novel displacement prediction model, the interaction mechanism is still not included, which limits the adaptability and comparability among different landslide cases. A better insight into the landslide development patterns is to be developed, combining the failure-mechanism-based evaluation method, in the future model for displacement prediction. Other than from the pure displacement prediction based on displacement, rainfall, and water level data sequence, an evaluation of the critical rainfall intensity and critical water level fluctuation rate is needed to be conducted under certain landslide development patterns in the further study.

In addition, landslide displacement is a noisy and non-stationary process that varies with time, which is highly affected by internal factors such as formation lithology and geological structure and external factors such as the rainfall, reservoir water level, and snow melting. Due to the complex nonlinear relationship between all these various inducing factors and landslide displacement, the landslide displacement prediction is subject to considerable uncertainties [56]. The limitations of the machine learning model, parameter selection, and data noise will increase the uncertainty of prediction [57]. The prediction model proposed in this paper is a deterministic point prediction model which cannot estimate the variability and uncertainty related to a given landslide displacement prediction, which limits its reliability under uncertain conditions. This should be addressed in the future study.

## 7. Conclusions

A reservoir landslide’s movement is closely associated with the related factors including reservoir level fluctuations, rainfall intensity, and previous deformations. The complex nonlinear relationship between all these various inducing factors and landslide displacement increased the challenge of forecasting in the form of considerable uncertainties. In this study, a novel prediction model for landslide displacement prediction was proposed to improve the accuracy by the combination of multiple algorithms. The EDR method can identify the most relevant factors influencing a landslide’s movements to use as input variables for an SVR model. The CEEMD method is suitable for the decomposition of various time series and can be used to extract the trend displacement of slow-moving landslide displacement. The CEEMD method can also highlight local fluctuations in the time series of related factors, and the frequency components of these time series can be extracted by combining the *t*-test method. With the help of MSI optimization algorithms, the optimal value of the penalty factor C and the kernel function parameter g for an SVR model can be obtained. This paper proposes an SVR model based on the CEEMD method, EDR selection, and MSI optimization algorithm that can capture the deformation characteristics of the landslide before failure.

Measurements of landslide displacements for the Shiliushubao landslide in the TGRA were used to demonstrate the novel displacement prediction model. The predicted displacements, including season fluctuations and the long-term trend, were found to be consistent with the observed data, which indicates that the proposed model has good predictive performance, even when the displacement characteristics are cyclic and complex. The DA- and GWO-based SVR model provided the best prediction of periodic displacement and trend displacement, respectively. The prediction model proposed in this paper has wider applicability. It can enhance the prediction of landslide displacements characterized by slow-moving, step-like displacements that are influenced by multiple related factors with frequency conversion characteristics.

## Figures and Tables

**Table 1:** Paired *t*-test results of all decomposed IMF.

Groups	Restructured Factor	Component	*t*	Sig.	Mean (mm)	Std. Deviation (mm)
Rainfall	L1	IMF_2_	0.22	0.83	1.50	56.92
		IMF_3_	−0.20	0.84	−1.23	50.75
		IMF_4_	0.60	0.55	3.02	42.24
		IMF_5_	0.10	0.92	0.41	36.49
	L2	IMF_2_	0.47	0.64	6.16	110.1
		IMF_3_	−1.34	0.18	−10.09	63.28
	L3	IMF_2_	0.23	0.82	3.05	111.1
		IMF_3_	−1.70	0.09	−12.42	61.56
		IMF_4_	−1.08	0.28	−6.75	52.48
	L4	IMF_2_	0.38	0.70	5.43	120.1
		IMF_3_	−0.61	0.54	−5.02	69.11
Reservoir water level	X1	IMF_2_	0.47	0.64	0.26	4.73
		IMF_3_	2.07	0.04	0.66	2.70
	X2	IMF_2_	0.22	0.83	2.91	111.6
		IMF_3_	−1.58	0.12	−11.65	62.23
		IMF_4_	−0.98	0.33	−6.13	52.64
	X3	IMF_2_	−0.17	0.86	−0.37	18.16
		IMF_3_	−0.52	0.61	−1.49	24.30
		IMF_4_	−2.19	0.03	−10.94	42.05
Displacement	D1	IMF_2_	−0.04	0.97	−1.16	229.4
		IMF_3_	−1.07	0.29	−40.82	320.5

**Table 2:** EDR distance between periodic displacements and related factors.

Groups	Component	Periodic Displacement			Trend Displacement
		Origin	High	Low	
Rainfall	L1	61	60	/	68
	L2	56	53	/	24
	L3	56	53	/	42
	L4	54	49	/	42
Reservoir level	X1	53	33	44	22
	X2	56	53	/	41
	X3	69	41	58	60
Displacement	D1	67	21	/	3
	D2	66	32	/	2

**Table 3:** Three benchmark functions.

Function	Range	Theoretical Minimum Value
$F_{1}\left(x\right)=\sum_{i=1}^{n}x_{i}^{2}$	$x_{i}\in \left[-100,100\right],\;i=1,\;2$	0
$F_{2}\left(x\right)=\sum_{i-1}^{n}ix_{i}^{4}+random\left(0,1\right)$	$x_{i}\in \left[-1.28,1.28\right],\;i=1,\;2$	0
$F_{3}\left(x\right)=\sum_{i=1}^{n}\left[x_{i}^{2}-10\cos\left(2\pi x_{i}\right)+10\right]$	$x_{i}\in \left[-5.12,5.12\right],\;i=1,\;2$	0

**Table 4:** Parameter and results of each optimization algorithm.

Algorithm	Parameters			Periodic		Trend	
				C	g	C	g
BA-SVR	Sizepop = 20	Max_iter. = 200	A = 0.2	220.67	0.00109	657.16	0.00106
	Lb = 1 × 10^−2^	Ub =1 × 10^2^	r = 0.5				
	Freq_min = 0.1	Freq_min = 0.2	Alpha = 0.2				
DA-SVR	Sizepop = 30	Max_iter. = 200	e = f = 0.1	66506	0.00001	83702	0.00001
	lb = 1 × 10^−5^	ub = 1 × 10^5^	c = 0.7				
	w = 0.5	s = 0.1	a = 0.1				
GOA-SVR	Sizepop = 30	Max_iter. = 200	l = 1.5	16.13	0.00100	29.68	0.01000
	lb = 1 × 10^−3^	ub = 1 × 10^3^	f = 0.5				
GWO-SVR	Sizepop = 30	Max_iter. = 200	dim = 2	474.94	0.00100	706.29	0.00100
	lb = 1 × 10^−3^	ub = 1 × 10^3^	/				
SSA-SVR	Sizepop = 30	Max_iter. = 200	pNum = 20%	16.17	0.00100	9677.9	0.00014
	lb = 1 × 10^−4^	ub = 1 × 10^4^	sNum = 20%				
OA-SVR	Sizepop = 20	Max_iter. = 200	dim = 2	1.74	0.01000	48277.4	0.00001
	lb = 1 × 10^−5^	ub = 1 × 10^5^	b = 1				

**Table 5:** Prediction accuracy of each SI in Baishuihe landslide.

Optimization Algorithm	Periodic Displacement				Trend Displacement			
	MAPE	RMSE	MAE	R^2^	MAPE	RMSE	MAE	R^2^
BA	0.688	13.691	30.118	0.757	0.395	1065.132	926.683	0.8621
DA	0.788	13.652	30.367	0.761	0.008	20.448	66.336	0.9997
GOA	0.692	13.663	30.110	0.758	0.008	19.649	66.214	0.9997
GWO	0.680	13.592	29.558	0.751	0.008	20.448	66.336	0.9997
SSA	0.786	13.589	30.307	0.762	0.009	22.766	64.733	0.9998
WOA	0.788	13.629	30.329	0.761	0.008	20.448	66.336	0.9997
